# Development of a clinical decision model for thyroid nodules

**DOI:** 10.1186/1471-2482-9-12

**Published:** 2009-08-10

**Authors:** Alexander Stojadinovic, George E Peoples, Steven K Libutti, Leonard R Henry, John Eberhardt, Robin S Howard, David Gur, Eric A Elster, Aviram Nissan

**Affiliations:** 1 Department of Surgery, Division of Surgical Oncology, Walter Reed Army Medical Center,Washington, D.C., USA; 2 Department of Surgery, Brooke Army Medical Center, Fort Sam Houston, TX, USA; 3 The United States Military Cancer Institute, Washington, D.C., USA; 4 Angiogenesis Section, Surgery Branch, National Cancer Institute, Bethesda, MD, USA; 5 Department of Surgery, National Naval Medical Center, Bethesda, MD, USA; 6 BioInformatics Division, DecisionQ, Washington, D.C., USA; 7 Department of Clinical Investigation, Division of Biostatistics, Walter Reed Army Medical Center, Washington, D.C., USA; 8 Department of Radiology, University of Pittsburgh, PA, USA; 9 Magee-Women's Hospital, Pittsburgh, PA, USA; 10 Department of Surgery, Hadassah-Hebrew University Medical Center, Mount Scopus, Jerusalem, Israel

## Abstract

**Background:**

Thyroid nodules represent a common problem brought to medical attention. Four to seven percent of the United States adult population (10–18 million people) has a palpable thyroid nodule, however the majority (>95%) of thyroid nodules are benign. While, fine needle aspiration remains the most cost effective and accurate diagnostic tool for thyroid nodules in current practice, over 20% of patients undergoing FNA of a thyroid nodule have indeterminate cytology (follicular neoplasm) with associated malignancy risk prevalence of 20–30%. These patients require thyroid lobectomy/isthmusectomy purely for the purpose of attaining a definitive diagnosis. Given that the majority (70–80%) of these patients have benign surgical pathology, thyroidectomy in these patients is conducted principally with diagnostic intent. Clinical models predictive of malignancy risk are needed to support treatment decisions in patients with thyroid nodules in order to reduce morbidity associated with unnecessary diagnostic surgery.

**Methods:**

Data were analyzed from a completed prospective cohort trial conducted over a 4-year period involving 216 patients with thyroid nodules undergoing ultrasound (US), electrical impedance scanning (EIS) and fine needle aspiration cytology (FNA) prior to thyroidectomy. A Bayesian model was designed to predict malignancy in thyroid nodules based on multivariate dependence relationships between independent covariates. Ten-fold cross-validation was performed to estimate classifier error wherein the data set was randomized into ten separate and unique train and test sets consisting of a training set (90% of records) and a test set (10% of records). A receiver-operating-characteristics (ROC) curve of these predictions and area under the curve (AUC) were calculated to determine model robustness for predicting malignancy in thyroid nodules.

**Results:**

Thyroid nodule size, FNA cytology, US and EIS characteristics were highly predictive of malignancy. Cross validation of the model created with Bayesian Network Analysis effectively predicted malignancy [AUC = 0.88 (95%CI: 0.82–0.94)] in thyroid nodules. The positive and negative predictive values of the model are 83% (95%CI: 76%–91%) and 79% (95%CI: 72%–86%), respectively.

**Conclusion:**

An integrated predictive decision model using Bayesian inference incorporating readily obtainable thyroid nodule measures is clinically relevant, as it effectively predicts malignancy in thyroid nodules. This model warrants further validation testing in prospective clinical trials.

## Background

Thyroid nodules represent a common problem brought to medical attention. Four to seven percent of the United States adult population (10–18 million people) has a palpable thyroid nodule(s), and up to 50% of American women older than age 50 have nodules visible by ultrasound [1]. The majority (>95%) of thyroid nodules are benign; however, malignancy risk increases with male gender, nodule size, rapid growth and associated symptoms, extremes of age (< 30 and > 60 years), underlying autoimmune disease, nodule growth under thyroid hormone suppression, personal or family history of thyroid malignancy and radiation exposure [2].

Thorough history and physical examination, serum thyrotropin (TSH) level, thyroid ultrasound (US) and fine need aspiration (FNA) comprise the standard evaluation of patients with thyroid nodules. Patients with thyroid nodules typically undergo both thyroid US and FNA. Nodules with maximum diameter > 1.0–1.5 cm with solid elements, or nodules demonstrating suspicious features on US particularly should undergo FNA [3]. Given the increased risk of malignancy in so-called thyroid incidentalomas detected by ^18^FDG-PET (14–50%) or sestamibi scan (22–66%), FNA is indicated under these circumstances as well [4,5].

Fine needle aspiration remains the most cost effective and accurate diagnostic tool for thyroid nodules in current practice. Although a standard of practice, FNA remains an imperfect diagnostic test for thyroid nodules, particularly when one considers the high frequency (>20%) of indeterminate cytology. A six tier classification system for FNA is favored that is associated with increased risk of malignancy across the spectrum of unsatisfactory or non-diagnostic FNA (unknown), benign (<1%), follicular lesion (atypia) of undetermined significance (5–10%), follicular neoplasm (20–30%), suspicious for malignancy (50–75%), malignant (100%) [3]. In experienced hands, sensitivity and specificity are very high, 95% and 99%, respectively, but sensitivity and specificity of FNA varies considerably, as it is highly dependent on the operator as well as the cytologist's skills [6,7]. In studies where cytology was compared to histology or revised by an expert cytologist, inaccuracy of the initial diagnosis was observed in up to 61% of the cases [8]. Unfortunately over 20% of patients undergoing FNA of a thyroid nodule have indeterminate cytology (follicular neoplasm) with associated malignancy risk prevalence of 20–30%, and they require thyroid lobectomy/isthmusectomy purely for the purpose of attaining a definitive diagnosis. Given that the majority (70–80%) of patients with "follicular neoplasm" has benign surgical pathology, thyroidectomy in these patients is conducted principally with diagnostic intent [9].

This emphasizes the need for non-invasive diagnostic imaging modalities with improved cancer detection accuracy coupled with clinically-relevant, treatment-directing malignancy risk prediction models to assist the clinician in the interpretation of available diagnostic information and minimize the frequency of purely diagnostic thyroid resections. We have previously studied the potential value of electrical impedance scanning (EIS) of thyroid nodules in a prospective feasibility trial. The overall diagnostic accuracy (73%) of EIS in that study was clinically meaningful, as utilization of the technology could result in a significant reduction (67%) in the number of purely diagnostic thyroid resections for cytologically determined follicular neoplasm [10].

Bayesian Belief Networks or Bayesian classification has gained acceptance as a methodology for characterizing multi-dimensional or complex data sets pursuant to developing disease risk prediction models [11,12]. A Bayesian Belief Network (BBN) is a graphical model that represents variables and their probabilistic independencies. Clinical observations such as symptoms, imaging data and lab results may be encoded into a BBN in order to estimate the probability of a disease or disorder [13,14].

Advances in machine learning allow users to train these networks on complex clinical problems using an intuitive computer program [15]. A BBN encodes the joint probability distribution of all the variables in the data set by building a directed acyclic network of conditional probabilities incorporating independent predictor nodes (variables), each with its own prior probability [11,16]. Conditional independence statements are embedded in the network structure through the arcs that connect the network's nodes [15]. These network arcs between nodes define a hierarchy and structure of information. Bayesian networks allow clinicians to derive insights about the data domain because the networks are graphical, hierarchical representations of how conditionally independent variables associate to inform a dependent outcome of interest, such as presence of malignancy. The inferential structure of the network allows the clinician to collect *a priori *evidence of independent variables, add this knowledge to the network and receive *a posteriori *probability of outcome.

We hypothesized that a Bayesian Belief Network analytical tool could be constructed using a machine learning platform applied to this specific patient study population represented by relevant clinical variables (e.g. patient age, gender, thyroid nodule size, US and impedance characteristics, and FNA cytology) in order to develop a model-derived risk assessment tool, which could support decision making on the basis of individual patient risk of malignancy. We further hypothesized that co-dependent analysis of EIS in the context of standard testing (US, FNA) would increase the utility of all of these studies through clinical decision support. The primary focus of this analysis is to determine the feasibility of a Bayesian predictive model to assist the clinician in interpreting diagnostic information.

## Methods

We trained a Bayesian classifier on a prospectively enrolled cohort [(n = 216; 110 with malignant thyroid nodules (51%)] collected over a four year period (Sept 2002 – Dec 2006) in the context of a previously published IRB-approved clinical trial including thyroid impedance, ultrasound imaging, cytological and histopathological outcome data [10,17]. This was a prospective single arm observational cohort trial evaluating the diagnostic accuracy of pre-operative thyroid EIS in patients scheduled to undergo thyroidectomy. Fifty percent of patients (n = 109) were undergoing diagnostic thyroidectomy for indeterminate FNA cytology. The objective was to train and validate a classifier that could be used for clinical decision making.

### Thyroid EIS Examination

Thyroid EIS was performed as described previously [10]. Thyroid EIS was conducted prior to thyroid surgery using the T-Scan 2000ED [TransScan Medical (Mirabel^®^), Austin, TX].

Impedance recordings of conductivity and capacitance were obtained over the entire gland in a predetermined sequence using a real-time image acquisition technique over a broad frequency range (frequency range, 50–20,000 Hz). A gray-scale impedance map provided an anatomical image corresponding to the area of interest directed to a palpable or sonographic thyroid nodule.

Homogeneous gray scale impedance maps (uniform conductivity and capacitance) are characteristic of normal or benign thyroid nodules, which demonstrate similar conductivity and capacitance (or impedance) to normal thyroid tissue. A focal disturbance in electrical field distribution by a malignant tumor due to its increased conductivity and, or capacitance (or decreased impedance) appears as a focal bright white spot on the gray scale impedance map. Changes from baseline sternocleidomastoid conductivity and capacitance were calculated for the thyroid nodule(s). A positive EIS examination was previously defined as a focal bright spot over a thyroid nodule correlating with increased conductivity (decreased impedance) and/or capacitance >25% baseline sternocleidomastoid muscle impedance, absent confounding local artifact [10,17].

For the purpose of this study two surgical oncologists (AS, AN) with extensive experience with EIS in general, and the T-Scan 2000ED in particular, performed critical review of impedance scans conducted in the previous trial, both blinded to fine needle aspirate cytology and surgical pathology results. They determined an EIS level of suspicion (LOS) score on the basis of a focal white spot presence and increased conductivity and, or capacitance (with the previously established 25% above baseline impedance cutoff) associated with the palpable or sonographic thyroid abnormality. Thyroid nodule Level of Suspicion was classified as follows in the blinded review: LOS 1: Definitely benign; LOS 2: Highly unlikely to be malignant; LOS 3: Unlikely to be malignant; LOS 4: Likely to be malignant; and, LOS 5: Highly likely to be malignant. Thyroid nodules corresponding to a palpable or sonographic abnormality determined to have LOS of 4 or 5 were considered EIS-positive; otherwise they were regarded EIS-negative.

All study subjects underwent thyroid resection after thyroid US, FNA and EIS. Surgical histopathology was correlated with sonographic, cytological and impedance findings and interpretations.

FNA and surgical specimens were evaluated by experienced board-certified cytologists and thyroid pathologists who rendered cytological and histopathological diagnosis without knowledge of EIS level of suspicion for malignancy.

### Statistical Analysis Plan Using Bayesian Belief Networks

Study data were collected and assembled into a data set consisting of 216 subjects, 109 with indeterminate cytology results. Biopsy results were classified based on established clinical guidelines into either Benign (n = 106) or Malignant (n = 110) diagnoses and assembled into a master data set. The master data set was then randomized into ten additional cross-validation sets. Each subject record was assigned a randomly generated number. These numbers were then used to assign the subjects to ten unique test groups. A unique training set consisting of the remaining 90% of cases was created for each test group.

Data analysis was then conducted using a Bayesian Belief Network (BBN). The BBN was built by applying a set of heuristics to generate predictive models with different conditional independence assumptions. The BBN we constructed encoded the joint probability distributions of all the variables in our clinical data set from our previously published clinical trial by building a network of conditional probabilities [17]. The BBN is a directed network incorporating parent-child relationships between nodes. The network was queried to provide estimates for posterior probabilities given *a priori *knowledge, and tested for accuracy using data withheld from the training model. The Bayesian network in this study was constructed using FasterAnalytics™, (DecisionQ, Washington, DC).

The network was validated using a train-and-test cross-validation methodology, in this instance ten-fold cross-validation. Cross-validation is an established technique in multivariate analysis which allows researchers to estimate the performance of predictive models when used outside of the research setting. This analysis calculates predictive values by classifying the outcome (surgical pathology diagnosis) for a given instance and comparing this prediction to the known value in an independent test set. The test set predictions were then used to calculate a receiver-operating characteristic (ROC) curve and inference matrix by threshold for each test set by clinical feature of interest.

The curve was calculated by comparing the predicted value for each feature of interest to the known value in the test set on a case-specific basis, rank-ordering the resulting predictions from most likely to least likely and calculating the curve using the assumption that the most likely cases would be evaluated first. This curve was then used to calculate area-under-the-curve (AUC), positive and negative predictive value (PPV and NPV).

## Results

**Figure 1:**
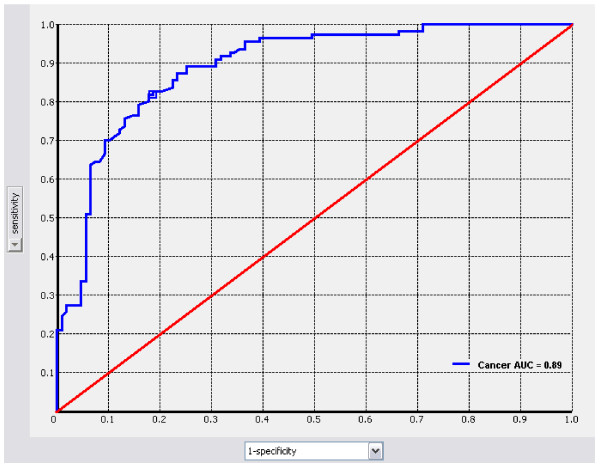
**ROC Curve for cancer prediction in validation against the master data**. Sensitivity is plotted on the y-axis and 1-specificity is plotted on the x-axis.

Clinical, image-based, cytological and pathological characteristics of the population are demonstrated in Table 1. Importantly the disproportionately high prevalence of indeterminate cytology is reflective of a referral-based population for operation enrolled in surgical clinics. These clinical data were encoded in a Bayesian Belief Network (BBN) in order to estimate the probability of thyroid nodule histopathology. As only very few patients in this cohort had a family history of thyroid cancer or exposure to radiation we elected not to include these parameters in the BBN. Figure 1 shows the ROC curve and area under the curve for the model tested *a posteriori *against the master data set of 216 patients for cancer detection, 110 with malignancy.

**Table 1:** Characteristics of the study population

**Patient Characteristics**	**No**.	**%**
Gender		

Male	46	21.3

Female	170	78.7

Patient age, years	mean ± SD = 47.1 ± 15.9	

Median (range)	47 (18 – 85)	
**Disease Characteristics**	**No**.	**%**

Serum TSH	mean ± SD = 2.6 ± 6.8	

Pre-operative thyroid status		

Euthyroid	174	80.6

Hyperthyroid	26	12

Hypothyroid	16	7.4

Dominant thyroid nodule size (cm)	mean ± SD = 2.8 ± 1.6	

Dominant thyroid nodule size		

<2 cm	78	35.9

2 – 4 cm	101	46.5

>4 cm	38	17.5
**Imaging Characteristics**	**No**.	**%**

*Thyroid Scintigraphy*		

Cold	21	58.3

Warm	3	8.3

Hot	12	33.3

*Thyroid Ultrasound*		

Simple cyst	4	1.9

Complex cyst	8	3.7

Mixed	32	14.8

Solid	172	79.6

*Thyroid EIS Level of Suspicion*		

I: Definitely benign	22	10.1

II: Highly unlikely to be malignant	49	22.5

III: Unlikely to be malignant	21	9.6

IV: Likely to be malignant	64	29.5

V: Highly likely to be malignant	62	28.4
**Pathological Characteristics**	**No**.	**%**

*Fine needle aspiration cytology*		

Inadequate	6	2.8

Not done	9	4.2

Negative	30	13.9

Positive	62	28.7

Indeterminate	109	50.4

*Surgical Histopathology*		

Benign	106	49.1

Malignant	110	50.9

The completed BBN was cross-validated using the training and test sets, and also tested *a posteriori *against the master data set to assess predictive power. Table 2 details the cross-validation results for each train-and-test pair and the *a posteriori *testing results. Cross validation of the model created with Bayesian Network Analysis effectively predicted malignancy [AUC = 0.88 (95% Confidence Interval (CI): 0.82–0.94)] in thyroid nodules. The positive and negative predictive values of the model are 83% (95%CI: 76%–91%) and 79% (95%CI: 72%–86%), respectively. Sensitivity and specificity of the model are 82% (95%CI: 74%–91%) and 77% (95%CI: 68%–86%), respectively, at a 50% threshold. In the cross-validation of the BBN model developed in this study, a 50% probability threshold (most likely case) for calling a case malignant or benign produced the highest results in this dataset (Table 2 and 3).

**Table 2:** BBN cross-validation results for each train-and-test pair and *a posteriori *testing results

	**AUC**		**PV @ 50%**		**Cancer, At 50% Threshold**		**Benign, At 50% Threshold**	
				
	**Benign**	**Cancer**	**Benign**	**Cancer**	**Sensitivity**	**Specificity**	**Sensitivity**	**Specificity**
Test 1	71.4%	71.5%	60.0%	66.7%	66.8%	50.0%	60.0%	58.3%
Test 2	93.4%	93.4%	80.0%	81.8%	81.8%	70.0%	90.0%	81.7%
Test 3	89.2%	88.7%	81.3%	85.7%	66.8%	85.7%	92.9%	55.6%
Test 4	89.8%	89.1%	81.8%	66.7%	90.0%	69.2%	77.1%	80.0%
Test 5	92.1%	92.2%	77.8%	91.7%	92.4%	87.5%	87.6%	77.0%
Test 6	99.8%	99.8%	100.0%	100.0%	100.0%	91.6%	100.0%	77.8%
Test 7	76.0%	76.0%	80.0%	80.0%	90.0%	80.0%	80.0%	70.0%
Test 8	90.6%	90.5%	78.6%	85.7%	66.8%	83.3%	100.0%	33.3%
Test 9	89.8%	90.5%	80.0%	81.8%	81.8%	70.0%	90.0%	72.7%
Test 10	88.2%	88.2%	70.0%	91.7%	85.6%	87.5%	87.6%	28.6%
								
**Mean**	**88.0%**	**88.0%**	**78.9%**	**83.2%**	**82.2%**	**77.5%**	**86.5%**	**63.5%**
								
Internal	88.6%	89.0%	81.5%	82.6%	81.8%	81.3%	82.4%	81.0%

**Table 3:** Contribution of first-order predictors (thyroid nodule size, US and EIS characteristics, and FNA cytology) to predictive power of the Bayesian model

	**AUC**		**PV @ 50%**	
		
	**Benign**	**Cancer**	**Benign**	**Cancer**
No EIS	84.2%	84.6%	75.8%	71.3%
No FNA	88.0%	83.0%	79.5%	82.9%
No US	88.2%	88.4%	80.4%	83.8%
No Nodule Size	92.0%	91.9%	83.5%	81.6%

**Figure 2:**
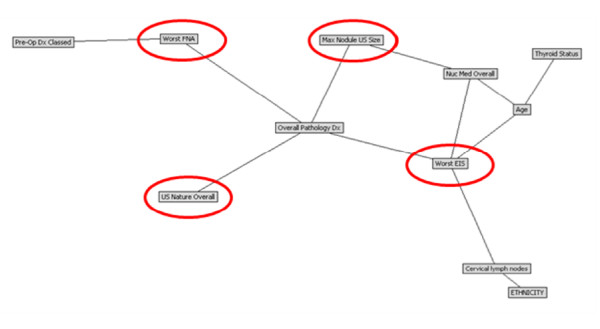
**Bayesian Belief Network model: Pathological diagnosis (Overall Pathology Dx) in thyroid nodules (Benign versus Malignant)**. The model structure defines four critical predictors of thyroid nodule histopathology (red circles): fine needle aspiration (FNA) cytology, maximum nodule size (determined by ultrasound), electrical impedance scan (EIS) and ultrasound (US) characteristics of the nodule. Worst EIS is based on LOS: I: Definitely benign; II: Highly unlikely to be malignant; III: Unlikely to be malignant; IV: Likely to be malignant; V: Highly likely to be malignant.

The resulting BBN is a directed graph of conditional dependence between variables. Figure 2 shows the structure of the BBN developed in this study to predict final histopathology in 216 patients with thyroid nodules. What we learn from the structure of the BBN is that four variables share direct conditional dependence with final histopathology: fine needle aspiration (FNA) cytology, maximum nodule size (determined by ultrasound), electrical impedance scan (EIS) and ultrasound (US) characteristics of the nodule. The relative contribution of each of these four factors was determined by excluding each factor one at a time in *a posteriori *analysis against the master data set of 216 patients. The only factor that significantly degraded the model, when eliminated from the network, was thyroid nodule EIS (Table 3). The features directly, and conditionally dependent with final histopathology were nodule size, ultrasound and EIS characteristics, and FNA cytology of the thyroid nodule. Further, the variables patient age, thyroid nodule size, scintigraphic findings (hot, warm, cold), and EIS characteristics are also conditionally dependent with one another and through thyroid nodule size and EIS characteristics inform final histopathology.

**Figure 3:**
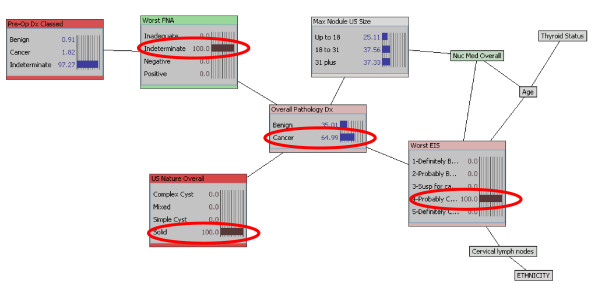
**Posterior estimate of surgical pathology outcome derived from prior knowledge of EIS result (EIS level of suspicion of 4 – likely to be malignant), ultrasound finding of solid thyroid nodule, and indeterminate FNA cytology**. Changing the EIS result from highly unlikely to be malignant (LOS 2) to Level of Suspicion of (likely to be malignant) increases the posterior probability of malignancy from 15% to 65% (red ellipses).

With a trained, tested, and cross validated model, the clinician can add evidence to the model given prior knowledge of a specific case through the selection of specific features and generate case-specific predictions of final histopathology. The final pathology diagnosis for a given patient with thyroid nodule EIS level of suspicion of 2 (highly unlikely to be malignant) has a posterior probability of cancer of 19%. Adding thyroid nodule ultrasound finding of 'solid' to the EIS level of suspicion of 2 refines the case specific posterior estimate of malignancy to 23%, which is less than the cancer rate in the study population. Additional data refines the prediction of malignancy even further; indeterminate FNA cytology of a 'solid' nodule by US, having EIS level of suspicion of 2 has a posterior probability of benignity of 85% (15% probability of malignancy). Changing the EIS result from highly unlikely to be malignant (LOS 2) to level of suspicion of 4 (likely to be malignant) increases the posterior probability of malignancy from 15% to 65% (Figure 3).

Inference-based individual case-specific estimates of posterior probability from the Bayesian Belief Network can also be developed by applying the model to new data sets in either batch inference mode or by tabulating all potential combinations in an inference table. Table 4 provides an example of an inference table calculated using the model developed in this study for all potential combinations of EIS and FNA cytology result, providing the clinician with a simple "look-up table" format which may be easier to interact with than the model. For example, the sixth case in Table 4, Definitely Benign EIS (Level of Suspicion of 1) and Indeterminate FNA cytology, has a probability of cancer of 5.7%. However, a patient with an indeterminate nodule with EIS Level of Suspicion of 4 (likely to be malignant) has a 58.7% probability of thyroid malignancy.

**Table 4:** Inference table calculated using the model developed in this study for all potential combinations of EIS and FNA result, selected subset.

**EIS Level of Suspicion**	**FNA Cytology**	**Surgical Pathology Diagnosis**	
		
		**Benign**	**Malignant**
1-Definitely Benign	Inadequate	100.00%	0.00%
2-Highly unlikely to be malignant	Inadequate	100.00%	0.00%
3-Unlikely to be malignant	Inadequate	100.00%	0.00%
4-Likely to be malignant	Inadequate	100.00%	0.00%
5-Highly likely to be malignant	Inadequate	100.00%	0.00%
**1-Definitely Benign**	**Indeterminate**	**94.30%**	**5.70%**
2-Highly unlikely to be malignant	Indeterminate	87.80%	12.20%
3-Unlikely to be malignant	Indeterminate	90.90%	9.10%
**4-Likely to be malignant**	**Indeterminate**	**41.30%**	**58.70%**
5-Highly likely to be malignant	Indeterminate	26.40%	73.60%
1-Definitely Benign	Negative	98.90%	1.10%
2-Highly unlikely to be malignant	Negative	97.60%	2.40%
3-Unlikely to be malignant	Negative	98.20%	1.80%
4-Likely to be malignant	Negative	79.70%	20.30%
5-Highly likely to be malignant	Negative	66.70%	33.30%
1-Definitely Benign	Positive	14.90%	85.10%
2-Highly unlikely to be malignant	Positive	7.10%	92.90%
3-Unlikely to be malignant	Positive	9.50%	90.50%
4-Likely to be malignant	Positive	0.70%	99.30%
5-Highly likely to be malignant	Positive	0.40%	99.60%

## Discussion

The primary aim of this study was to develop a Bayesian Belief Network model based on data collected prospectively in the context of a clinical trial evaluating the feasibility of electrical impedance scanning in patients with thyroid nodules pre-determined to undergo thyroid resection. Relevant clinical variables were included in the model in order to develop a model-driven risk assessment tool, which could support decision making on the basis of individual patient risk of malignancy. The model created with Bayesian Network Analysis effectively predicted malignancy [AUC = 0.88 (95%CI: 0.82–0.94)] in thyroid nodules. The positive and negative predictive values of the model are 83% (95%CI: 76%–91%) and 79% (95%CI: 72%–86%), respectively.

The thyroid nodule is a prevalent clinical problem in the United States, and the majority of nodules are pathologically benign. The increasingly frequent use of sensitive diagnostic modalities has contributed to an unprecedented rise in the incidence of differentiated thyroid carcinoma [18]. The preponderance of identified papillary thyroid cancer is small sub-clinical, indolent disease; hence, the challenge to the clinician is differentiating tumors of favorable biology from those with notoriously aggressive behavior [18]. Although clinical indicators of malignancy risk can facilitate therapeutic decision making, they are imperfect in directing treatment for those patients most likely to benefit from thyroidectomy. Another vexing, more fundamental problem than defining biology of malignancy, is that of definitively diagnosing the cytologically indeterminate thyroid nodule. This often necessitates diagnostic operation in a large proportion of patients with so-called "follicular neoplasms", to benefit possibly the few patients that actually have thyroid malignancy. Accurately predicting malignancy in any given thyroid nodule remains a daunting clinical challenge, establishing the need for decision support tools or predictive models to guide therapeutic decision making. The present study implemented Bayesian classification on a prospectively enrolled clinical trial cohort including clinical, image-based as well as cytological predictors of malignancy. A clinically relevant prognostic risk assessment tool was constructed and cross validated, which provides individual patient-specific prediction of malignancy in thyroid nodules.

Bayesian classification has been applied across the spectrum of medicine [19,20] from optimization of pharmacotherapy dosing [21,22], predicting cancer screening [23] and diagnostic test results [24,25], to determining injury severity [26] and ICU mortality [27], assessing operative risk [28] and predicting surgical outcomes [29-32]. More recently, BBN models have been developed to predict cancer-specific outcomes [33-37]. The findings of the current study demonstrate that the BBN model provides an individualized estimate of cancer risk in thyroid nodules, in three clinically relevant categories of FNA cytology category: negative, positive, and indeterminate. The receiver operating characteristic curves can be used to optimize the model for negative and positive predictive value in our thyroid cohort. Importantly, a patient in our broad population with an indeterminate nodule with EIS Level of Suspicion of 4 or 5 (likely or highly likely to be malignant) has a 58.7% and 73.6% probability of thyroid malignancy, respectively, according to the prognostic risk assessment tool developed and cross-validated herein. The predictive model developed in this study not only provides an individualized estimate of risk of malignancy in patients with a broad spectrum of thyroid nodules, it also can support integration with clinical systems (electronic health record) and provide real time estimates of risk, thereby facilitating clinical decision making and patient education. The iterative nature of the modeling methodology permits addition of new data, which can be used to update, or re-train and validate, dynamically modify and optimize the model. Model optimization with new data input over time will be important, as patients with indeterminate FNA cytology and EIS level of suspicion ranging from 1–3 (Normal to Unlikely to be malignant) in the current model have a clinically meaningful (~10%) likelihood of malignant histopathology.

There are several limitations inherent in the prognostic risk assessment tool constructed in this study. Other clinical data such as ^18^F-FDG Positron Emission Tomography, Doppler ultrasound, Magnetic Resonance Imaging and quantitative RT-PCR assays for thyroid-cancer-related genes of fine needle aspirates, which were not tested in this clinical trial, may be relevant and could improve the predictive value of the model.

Ultrasound variables considered in the BBN model development included primary thyroid nodule characteristics (e.g. solid versus cystic) and maximum dimension; however, other sonographic variables not measured in the study could have incremental predictive value, including color Doppler ultrasound-directed qualitative intra-nodular vascular distribution and microcalcifications, as well as quantitative analysis of tumor vascularity (tumor vascular resistive index). Although elimination of thyroid nodule impedance characteristics from the network significantly degraded the model in this study, thyroid impedance remains investigational and warrants further clinical validation. Further, while the predictive model was cross validated to assess robustness, it remains to be independently and prospectively validated in a new and expanded diverse patient population with thyroid nodules. This will be particularly important recognizing another putative factor limiting the generalizability of our study results – the selected pre-operative, disease-enriched population. The increased prevalence of disease biases the estimates of the positive predictive value (overly optimistic) and negative predictive (overly pessimistic). The ultimate value of the model will rest in its ability to predict malignancy in a general population of patients with thyroid nodules, where the prevalence of malignancy is decidedly lower. Importantly, we anticipate that the validated model will be utilized in situations of clinical uncertainty after standard testing (US and FNA) in order to facilitate clinical decision making with respect to operative indication.

Recognizing that individual variables, though independently associated with thyroid cancer, are insufficient in predicting of risk of malignancy in any given thyroid nodule, other investigators have stressed the importance of developing multivariate predictive algorithms to determine cumulative risk of malignancy for this common clinical problem [38,39]. Raza et al. utilized a multivariate stepwise regression model to predict malignancy in thyroid nodules in a highly selected patient population on the basis of patient age, calcifications in a sonographically solid nodule, and FNA cytology [39]. Tuttle, Lamar and Burch applied multivariate modeling in patients with indeterminate thyroid nodules to define male gender, nodule size exceeding 4 cm, and character of the gland by palpation (dominant nodule in multi-nodular goiter) to predict risk of thyroid malignancy [38]. Their analysis was limited to a narrow population of patients with follicular neoplasia by FNA, and did not include any imaging-based variables in the predictive model.

## Conclusion

Our study is in agreement with these investigations in that it suggests that a broad statistically validated network structure of multiple clinical variables has the potential to provide a universal method to individualize patient care. The dynamic, quantitative case-specific predictions made by this type of a predictive model could allow clinical decision support tools to be adapted to the specific needs and capabilities of a given medical clinic. This preliminary yet promising clinical tool clearly warrants further validation testing in planned prospective trials. If prospective validation of the model is successful we anticipate the model to serve as a web-based clinical tool, which can be accessed by physicians, and utilized by them in order to evaluate the risk of malignancy in individual patients presenting with thyroid nodule(s).

## Abbreviations

**BBN**: Bayesian Belief Network; **EIS**: Electrical Impedance Scanning; **ICU**: Intensive Care Unit; **F-FDG PET**: Fluorodeoxyglucose Positron Emission Tomography; **FNA**: Fine Needle Aspiration; **NPV**: Negative Predictive Value; **PPV**: Positive Predictive Value; **ROC**: Receiver Operating Characteristics; **RT-PCR**: Real Time Polymerase Chain Reaction; **TSH**: Thyrotropin.

## Competing interests

The authors declare that they have no competing interests.

## Authors' contributions

All authors read and approved the final manuscript

AS concived and designed the project, aquired, analysed and interpreted the data with statistical expertise, drafted and made critical revisions to the manuscript, obtained funding and supervied the overall project.

GEP aquired the data, made crittical revisions to the manuscript, and supervised the project.

SKL analysed and interpreted the data, made critical revisions to the manuscript and supervised the project.

LRH made crittical revisions to the manuscript.

JE concieved and designed the project, analysed and interpreted the data with statistical expertise, drafted and made critical revisions to the manuscript.

RSH analysed and interpreted the data with statistical expertise.

DG obtained funding, supervised the project and made crittical revisions to the manuscript.

EAE made critical revisions to the manuscript.

AN aquired the data, drafted the manuscript, made critical revisions to the manuscript, obtained funding, and supervised the project.

## Pre-publication history

The pre-publication history for this paper can be accessed here:


